# Asthma education material for children and their families; a global survey of current resources

**DOI:** 10.1186/s40413-015-0084-x

**Published:** 2015-12-14

**Authors:** Mark L. Everard, Ulrich Wahn, Sofia Dorsano, Elham Hossny, Peter Le Souef

**Affiliations:** School of Paediatrics and Child Health, University of Western Australia, TelethonKids Institute, Princess Margaret Hospital for Children, Roberts Road, Subiaco, 6008, Western, Australia; Department of Pediatric Pneumology and Immunology, Charité Humbolt University, Berlin, Germany; World Allergy Organization, International Headquarters, Milwaukee, Wisconsin United States; Pediatric Allergy and Immunology (PAI) Unit, Children’s Hospital of Ain Shams University, Cairo, Egypt

**Keywords:** Asthma, Children, Education

## Abstract

One of the keys to high quality paediatric asthma management is the provision of age appropriate information regarding the disease and its management. In order to determine whether the generation of a minimum dataset of information which can be translated into a wide range of languages might be used to assist children and their parents around the world, we undertook a survey of national Member Societies of the World Allergy Organization (WAO) to determine what educational material on asthma for children and their families already exists.

A questionnaire was developed using Survey Monkey and distributed in 2014 to 263 representatives of the WAO member Societies from 95 countries.

Thirty-three replies were received from thirty-one countries. The survey highlighted a considerable disparity in availability of material among the responding countries, with some countries reporting that information was freely available in hard copy and online and others reporting a lack of suitable material locally.

The results highlight the need to develop a core set of simple, clear and consistent age appropriate information that can be easily translated and delivered in a cultural and educationally effective format.

## Background

Asthma remains the commonest chronic disease of childhood and is associated with high levels of morbidity [1–6]. The World Health Organisation (WHO) has estimated that there are 300 million individuals with asthma worldwide with a projected growth of a further 100 million over the next decade [4]. A high proportion of these asthmatic subjects are children. Although the prevalence varies from region to region and country to country [1–5], the majority of individuals with asthma live in developing countries. The severity of the condition varies from occasional relatively mild symptoms to frequent troublesome symptoms that have a dramatic impact on quality of life and are associated with life threatening attacks. Indeed, WHO has estimated that asthma is responsible for 250,000 deaths per annum worldwide [4]. Currently, there is no cure for asthma, but there is good evidence that drugs such as inhaled corticosteroids [ICS], which have been available for more than 40 years, can transform the lives of most patients with symptoms of sufficient frequency and/or severity to require regular ‘preventer’ therapy. However, in order to reap the benefit of these drugs in terms of improved quality of life, protection against day to day bother, night time wakening, missed school or work due to exacerbation and indeed reduced mortality, the patient is required to use the ICS aerosol delivery system effectively every day [5, 7] - something that is rarely achieved. Consequently unnecessarily high levels of morbidity and mortality continue to be reported from countries around the globe both in developing [4–6, 8] and developed countries [9–11].

Although the basics of good asthma care generally involve doing the simple things right; that is, ensuring the diagnosis is correct, identifying any co-morbidities that may be exacerbating or mimicking asthmatic symptoms and, in those with sufficient symptoms to warrant a preventer therapy, ensuring the medication is taken regularly and effectively. Getting this right has been shown to yield huge benefits for patients and for healthcare systems with reduced hospitalisations, unscheduled health care contacts and deaths. However, there are many impediments to translating the potential of current interventions into real life as outlined by many authors and organisations, and most are relevant to a greater or lesser degree in all health care settings. These included lack of availability of medication, cost of medication, lack of awareness of the nature of the disease in the community, lack of an appropriate informed and knowledgeable cohort of health care professionals, lack of access to health care professionals and lack of knowledge linked to self-management among patients and their families.

A number of countries have developed National Asthma Plans [12, 13] to address many of these issues and report significant success in terms of improved outcomes for patients and reduced health care costs for the providers. One aspect of all such plans is the provision of cultural and age appropriate specific information for the patient and their family linked to a treatment plan and self-management. This echoes current beliefs about the optimal approaches to the management of chronic diseases in general as well as current recommendations in national and international guidelines for the management of asthma (http://www.ginasthma.org/), (http://www.sign.ac.uk/pdf/SIGN141.pdf). In some European countries such as Germany, pediatric asthma schools have become an essential part of outpatient care and are reimbursed by the National Health Insurances. In others, there are valuable resources available at a national and/or local level which can be used by health care professionals in developing a partnership with the patient, or accessed by children and families independently via the internet. However in many countries, there are no such resources or if they exist they are only available at certain centres.

Based on a perceived need, the World Allergy Organization (WAO) Committee on Pediatric Asthma proposed that consideration should be given to the development of a WAO educational program for pediatric asthma that could be tailored to the needs of different regions and countries. The objectives of the WAO program would be: (1) To improve the quality of life of asthmatic children, provide maximum autonomy in spite of the chronic disease, and optimize the available treatment options for children. (2) Ensure that no matter where children diagnosed with asthma grow up, they and their caregivers will receive the same degree of minimum education. The aim of this study was to identify the types of educational material available around the world and to determine whether a core multi-lingual resource available to health care professionals and patients would be valuable.

## Methods

A questionnaire was developed using the Survey Monkey application and distributed to 263 individuals from 95 WAO Member Societies around the world. The survey sought information regarding the availability and quality of educational material aimed at children and parents available in that country and whether material was available online.

## Results

Thirty-three replies were received from 31 countries, as documented in Table 1. The distribution of responses included 11 countries in Europe, 8 in South America, 3 in the Indian sub-continent, 3 in the Middle East, 2 in Africa, 2 in the Far East and one each from North America and Australasia.

**Table 1:** Summary of survey responses

Country	Are there national bodies producing asthma educational material and/are they of high enough quality?	Accessible via web?	Do you use this material?	Is there evidence for its effectiveness?
Argentina	Yes/Yes	Google, Medline, etc.	Yes	Yes insufficient
Australia	Yes/Yes	Nationalasthma.org.au	Yes	Yes unsure what
Austria	Yes/Yes	No	Yes	Yes for some issues
Bangladesh	Yes/No	Hard copy only	Yes	Yes
Belarus	Yes/Yes	Yes	Yes	Yes
Brazil	No	-	-	-
Canada	Yes/Yes Yes/Yes	www.asthma.ca/ www.aaia.ca [Eng & French] www.lung.ca	Yes No	No No
Croatia	Yes/Yes	asjastipic90@gmail.com	Yes	Yes
Cuba	Yes/Yes	www.sld.cu/sitios/asma/ www.infomed.sld.cu	Yes	No
Denmark	No	- pharma prod material is available	-	-
Ecuador	No	From AAAAI and WAO	-	-
Egypt	Yes/No	www.espai-eg.org/espai.htm	Yes	No
Greece	No	-	-	-
Portugal	Yes/Yes	Hard copy only	Yes	Yes a little
India	No Yes/Yes	Is pharma prod material Yes	- -	- -
Iran	Yes/Yes	Iranian Society of Asthma & Allergy (ISAA)	Yes	No
Japan	Yes/Yes	Yes	Yes	Yes
Kuwait	Yes/No	No	Yes	Yes
Latvia	Yes/No	yes	Yes	No
Lebanon	No	- Is pharma prod material	-	-
Panama	Yes/No	No	No	Yes
Sri Lanka	Yes/No		No	No
Sweden	Yes/Yes	http://astmaoallergiforbundet.se	No	Yes
Switzerland	Yes/Yes	http://www.aha.ch/swiss-allergy-centre/info-on-allergies/?oid=1444&lang=en	Yes	No
Turkey	Yes/No	No	Yes	Yes
Uruguay	No	No Use AAAAI Spanish info	Yes	Yes
UK	Yes/Yes	http://www.asthma.org.uk/advice www.asthma.org.uk/knowledge-bank	Yes	No
Venezuela	Yes/No	No	-	-
Zimbabwe	No	-	-	-

The availability of patient educational material varied from those reporting that printed information was widely available in both hard copies and on the Internet to others who report no specific educational material being available. Among the comments made, many reported that when information was available in printed form in a country its availability was limited due to poor distribution and access. Others commented on the lack of age appropriate material, lack of time to participate in the educational process, the absence of material designed to address cultural, and educational aspects of patient understanding of their disease. In a search of available literature in the English language a number of resources with translations were found.

## Discussion

The results from this survey, while limited to only 31 countries, highlight the significant disparities regarding the availability of educational material aimed at children with asthma and their parents across the globe. As such, it highlights the opportunity for creating a core educational resource that can then be developed for specific languages taking into account local cultural issues. The information referred to by the respondents to this survey is almost all written with some illustration, a further limitation for its use in many settings. With the exponential increase in mobile phone use around the world as well as the prevalence of the Internet, the focus of any developments in this area should probably be in the field of downloadable “apps” and online developments which would potentially address issues of literacy and cultural disconnect.

There is an extensive literature regarding the use of education in influencing health care outcomes in those with chronic diseases. It appears in many health care settings that the provision of information alone is, in the majority of cases, not sufficient to change health care beliefs and behaviour. The key aspects of the “educational” message and the format in which it is presented that do make a difference have not been clearly identified. Where change has been effective the education has been part of a greater package which includes improved knowledge among health care professionals delivering the care, greater accessibility to health care professionals with specific expertise, more frequent structured contact between the health care system and patient, a focus on a partnership approach to health care and practical issues such as addressing the cost burden for patients [14–19]. The greatest benefits for patients appear to be gained where a national structured approach is adopted. Such National Asthma Plans are unlikely to be rolled out in many countries due to lack of resources, considerations that asthma is a low priority in the context of that country or in-built institutional issues or self-interest. In the absence of a National Plan, the provision of opportunistically providing education to those presenting to emergency departments or admitted to hospital have been shown to reduce subsequent presentations and hospitalisations. Furthermore, while there are questions about the effectiveness of information alone in changing behaviour, the inability to readily access simple, clear and accurate information in an appropriate format places the majority of subjects with asthma at great disadvantage.

## Conclusion

The results of this international survey highlight the need to develop a core set of simple, clear and consistent age appropriate information that can be easily translated and delivered in a cultural and educationally appropriate format. The use of mobile phones and the internet allowing the presentation of both visual and auditory information, together with resources of pictorial action plans, has the potential to greatly enhance the reach of the information to population currently largely excluded. Increased provision of these educational resources can support the goals of improved paediatric asthma management.
